# Hantavirus Research in Finland: Highlights and Perspectives

**DOI:** 10.3390/v13081452

**Published:** 2021-07-26

**Authors:** Antti Vaheri, Heikki Henttonen, Jukka Mustonen

**Affiliations:** 1 Department of Virology, Medicum, University of Helsinki, 00290 Helsinki, Finland; 2 Wildlife Ecology, Natural Resources Institute Finland, 00790 Helsinki, Finland; ext.Heikki.Henttonen@luke.fi; 3 Department of Internal Medicine, Tampere University Hospital, 33520 Tampere, Finland; jukka.mustonen@tuni.fi; 4 Faculty of Medicine and Health Technology, Tampere University, 33014 Tampere, Finland

**Keywords:** bank vole, ecology, hantavirus, hemorrhagic fever with renal syndrome, nephropathia epidemica, Puumala virus, rodent

## Abstract

Finland has the highest incidence of hantavirus infections globally, with a significant impact on public health. The large coverage of boreal forests and the cyclic dynamics of the dominant forest rodent species, the bank vole *Myodes glareolus,* explain most of this. We review the relationships between Puumala hantavirus (PUUV), its host rodent, and the hantavirus disease, nephropathia epidemica (NE), in Finland. We describe the history of NE and its diagnostic research in Finland, the seasonal and multiannual cyclic dynamics of PUUV in bank voles impacting human epidemiology, and we compare our northern epidemiological patterns with those in temperate Europe. The long survival of PUUV outside the host and the life-long shedding of PUUV by the bank voles are highlighted. In humans, the infection has unique features in pathobiology but rarely long-term consequences. NE is affected by specific host genetics and risk behavior (smoking), and certain biomarkers can predict the outcome. Unlike many other hantaviruses, PUUV causes a relatively mild disease and is rarely fatal. Reinfections do not exist. Antiviral therapy is complicated by the fact that when symptoms appear, the patient already has a generalized infection. Blocking vascular leakage measures counteracting pathobiology, offer a real therapeutic approach.

## 1. Introduction

Hantavirus diseases are currently classified into two categories: hemorrhagic fever with renal syndrome (HFRS) and hantavirus cardiopulmonary syndrome (HCPS), although hemorrhages also occur in HCPS, and the heart and lungs are also affected in HFRS. Taxonomically human-pathogenic hantaviruses are members of the *Orthohantavirus* genus. However, in the following, we refer to them using the simple designation “hantaviruses”. Nephropathia epidemica (NE) is a hemorrhagic fever; the clinical picture is dominated by acute hemorrhagic tubulointerstitial nephritis. NE was first described in Sweden and Finland, but it is now known to occur in several other European countries, including Russia. The first detailed description of the clinical features of NE came from Finland [1].

It is of historical interest that a NE-like disease was encountered in both German and Finnish troops in Eastern Finnish Lapland starting in April of 1942 [2]. Our conclusion is that the epidemic was caused by Puumala hantavirus (PUUV).

PUUV, the only known hantaviral human pathogen in Finland [3], has a significant impact on public health [4]. The reported average incidence of diagnosed PUUV infections in Finland is 31 to 39 cases per 100,000 inhabitants [5,6,7]. From the seroprevalence up to 12.5% in the adult population [7] and the incidence, it has been calculated that only 20%–30% of infected humans seek medical attention leading to serological confirmation [4]. Most of the hospitalized NE patients have acute kidney injury (AKI), and up to 6% of them require transient dialysis treatment. The case fatality rate is very low, ranging from 0.08% to 0.4% [4].

Because of the high incidence of NE in the Finnish population, much research has been invested, in addition to NE epidemiology, virology, and clinical course, also into the ecology of virus in the carrier species, the bank vole *Myodes glareolus*. We review here the main fields and results of hantavirus research in Finland. On several topics, there are original and review articles in this Special Issue.

### 1.1. Discovery of Puumala Virus

In the late 1970s, Ho Wang Lee and Karl Johnson had detected a specific serologic response in patients with Korean hemorrhagic fever using lung tissue of striped field mice (*Apodemus agrarius*) from endemic areas as antigen [8,9]. Using a similar approach Markus Brummer-Korvenkontio and Antti Vaheri detected a specific serological response in NE patients using lung tissue of bank voles (today designated *Myodes glareolus*) and an immuno-fluorescence antibody test (IFAT) [10]. It was soon observed that many individuals in Finland had antibodies to the NE virus without any history of clinical disease [6].

Newly discovered viruses are usually named according to the site of discovery. In passing, it may be noted that we had two alternatives, Puumala and Pieksämäki, and chose the former, considering the obvious difficulties that the English-speaking community would have had with the latter. PUUV causing NE-type HFRS formed a distinct antigenic and genetic group in the Hantavirus genus. The adaptation of hantaviruses, including PUUV, to Vero E6 cells represented a major advance for research [11], also for diagnostics [12].

Incidentally, it may also be noted that the residents or municipal directors of the Puumala community in Eastern Finland have never objected to the designation Puumala virus as a pathogen. This may be compared to the pathogen Sin Nombre virus first detected in SW USA that did not receive a name according to the site of original discovery.

### 1.2. Development of Specific Diagnosis of PUUV-HFRS (NE)

Before serological diagnostics of NE, renal biopsies were often performed for diagnostic purposes. The finding of acute hemorrhagic tubulointerstitial nephritis was a specific characteristic finding. From 1979 to 1982, the diagnosis was IFAT on acetone-fixed lung sections of infected bank voles, >4 increase in IgG titer. Routine diagnostic service was started in Finland in 1981, and the use of acetone-fixed PUUV-infected Vero E6 was initiated in 1983. An immunochromatographic IgM assay as a point-of-care (POC) test was launched in Finland in 2003 [12]. Today, IgM enzyme immunoassay and POC tests are in general use in Finland. In Finland, 1000–3300 cases of hantavirus disease are diagnosed annually, the highest incidence globally. For sero/genotyping of hantavirus infections in humans and carrier rodents, neutralization tests and RNA sequencing are required.

Non-invasive rapid diagnosis of NE is feasible even from urine by demonstration of antigen-specific immunoglobulin light chains [13].

### 1.3. Immune Response and Pathogenesis

Hantaviruses are carried by rodents, insectivores, and bats in which they cause persistent and generally asymptomatic infections. This is also true for PUUV in bank voles (*Myodes glareolus*) [3].

PUUV does not cause direct cytopathology (cell death) or apoptosis but induces a vigorous immune response, both B and T cells. Human CD8 T cell response in PUUV infection occurs after the acute phase and, curiously, is associated with boosting of EBV-specific CD8 memory T cells [14] and nonrelated toxoid immunity: tetanus-specific and pertussis-specific IgG as well as T cells [15]. Regulatory T cell response in NE is also prolonged and correlates with disease severity [16]. Coinfection of PUUV with other rodent-borne virus infections (lymphocytic choriomeningitis, Ljungan, orthopox) does not have distinct effects on the course of NE [17].

A notable feature is that PUUV infection of vascular endothelial cells increases their permeability, which may result in vivo in vascular leakage, edema in many tissues, and decreased blood pressure. This is likely to explain our success in the use of icatibant (a bradykinin receptor antagonist) in the therapy of severely ill NE patients [18,19,20].

In addition to vascular leakage, excessive complement activation is another feature of severe and lethal NE [21,22,23]. However, there are many poorly understood features in NE pathobiology such as (i) the role of galectin-3-binding protein, the levels of which correlate with increased complement activation and with clinical variables reflecting the severity of acute hantavirus infection [24], (ii) the mechanism of thrombocytopenia [25], (iii) neutrophil activation [26], and (iv) interferon-dependent induction of tissue plasminogen activator (tPA) both in cultures of endothelial cells and in vivo in NE patients [27]. We suggested that tPA may be a general factor in the immunological response to viruses.

### 1.4. Genetic Factors in the Pathogenesis

Our pioneering works showed that HLA-B*08 and DRB1*0301 are likely to have the most severe form of the PUUV infection with lower blood pressures, more severe thrombocytopenia, and acute kidney injury (AKI) [28,29]. These were the first reports where a certain HLA haplotype is found to be associated with the clinical course of an acute viral disease or acute nephritis. The same HLA haplotype also associates with more virus excretion into the urine and into the blood [30].

On the contrary, individuals with HLA-B*27 have a benign clinical course [31]. Since these findings, several other class I and class II HLA haplotypes have been linked with severe or benign hantavirus infections, and these haplotypes varied among localities and hantaviruses. In addition, genetic variation in cytokines may affect the outcome of HFRS [32].

### 1.5. Immunogenetic Factors in Rodents

The comparison of genetic differentiation estimated between bank vole populations sampled over Europe, including Finnish samples, evidenced signatures of selection for Tnf, Mx2, and the Drb Mhc class II genes. Altogether, these results corroborated the hypothesis of the evolution of tolerance strategies in rodents [32,33].

Coinfections with pathogens and parasites are a rule in nature. T helper-1 immune pathway is involved principally in response to intracellular infections (such as viruses), and the T helper-2 immune pathway regulates infections with extracellular parasites, such as helminths. Pathway 2 can affect the processes in the first one. Guivier et al. [34] demonstrated a negative correlation between PUUV load and the expression of the Tnf-a and Mx2 genes in bank voles. A nematode *Heligmosomum mixtum* is widely spread in bank voles in Europe and is very common also in Finland [35]. TNF-α was more strongly expressed in voles infected with PUUV than in uninfected voles or in voles co-infected with *H. mixtum* and PUUV. *H. mixtum* may limit the capacity of the vole to develop proinflammatory responses [34]. This effect may increase the risk of PUUV infection and replication in host cells. This suggests that coinfections and immune gene expression may shape PUUV epidemiology.

## 2. Phylogeography and Microevolution

It has been suggested that in Germany, the absence of PUUV in the northeast part of the country could be due to different mt-DNA lineages of the bank vole [36]. However, in Finland, PUUV eastern lineage is common in bank voles irrespective of the mt-DNA lineage of the host, be it eastern lineage in S Finland or Ural lineage in N Finland. On the other hand, in western Lapland, N Finland, close to the Swedish border, there is a south-north contact zone of eastern and North-Scandinavian PUUV lineages in the middle of the range of the Ural lineage of the bank vole [37]. Furthermore, within this contact zone of eastern and North-Scandinavian PUUV lineages, frequent reassortment between the two lineages was found.

PUUV microevolution was studied throughout a host population cycle that covered two peak phases and two population declines [38]. Analyses of these PUUV variant sequences disclosed the following patterns: (1) nucleotide substitutions are mostly silent and deduced amino acid changes are mainly conservative, suggesting stabilizing selection at the protein level, (2) the three genome segments accumulate mutations at different rates, (3) some of the circulating PUUV variants are frequently observed while others are transient, (4) frequently occurring PUUV variants are composed of the most abundant segment genotypes (copious) and new transient variants are continually generated, (5) reassortment of PUUV genome segments occurs regularly and follows a specific pattern of segments association, (6) prevalence of reassortant variants oscillates seasonally and is higher in the autumn than in the spring, and (7) reassortants are transient, i.e., they are not competitively superior to their parental variants. These observations support a quasi-neutral mode of PUUV microevolution with a steady generation of transient variants, including reassortants, and preservation of a few preferred genotypes.

### 2.1. Ecology and Epidemiology of PUUV and NE in Finland

#### 2.1.1. The Historical Salla Epidemic

The famous epidemic in Salla during WW2 in eastern Lapland in 1942 has aroused speculation for decades [2]. Less than 100 Finnish and more than 1000 German soldiers were sick. The year 1942 was a cyclic peak phase of the vole cycle in Lapland. Bank voles (called “mice” in contemporary medical reports) were common in trenches. There are no mice so far north in Finland. This year was also characterized by the high density of Norway lemmings (*Lemmus lemmus*) [39], which has added flavor to speculations. However, we have not found a hantavirus in *Lemmus* in Fennoscandia ([40] and later unpublished data). Neither were we able to infect *Lemmus lemmus* by PUUV [41], which rejects the possibility of a host switch from bank voles to lemmings during the epidemic.

In the 1990s, a specific group of Finnish veterans known to have been sick during the epidemic in Salla in 1942 was sampled. They were hantavirus seropositive, but neutralization tests could not distinguish between *Myodes* virus carried PUUV and Topografov virus (TOPV) isolated from *Lemmus sibiricus* from Taimyr Peninsula [40]. The human antibodies had lost their specificity and were too old for neutralization tests. Concluding, because a lemming hantavirus has not been found in Fennoscandia, our conclusion is that PUUV from bank voles caused the Salla epidemic. This conclusion has sometimes been questioned because the timing of the epidemic was not typical; it occurred from April to late summer. Normally the epidemic peak is in late autumn–early winter [42]. However, according to the cyclic pattern, after the increase in the previous year, the bank vole populations were high already in spring 1942, and reports mentioned the abundance of “mice” (=bank voles) in the trenches.

#### 2.1.2. PUUV and Bank Vole Dynamics in Finland

In the boreal taiga of Northern Europe, including Finland, bank vole populations fluctuate strongly in a 3-to-4-year pattern [43,44,45], and the NE epidemics are related to the cyclic increase and peak years of the bank voles [46]. Immediately after the discovery of PUUV, the first longitudinal monitoring of PUUV in bank voles was started in a highly PUUV-endemic region in Central Finland [6]. These were the first data on the dynamics of any hantavirus in a fluctuating rodent host population. Those data already suggested, as well as later confirmed [46,47], that the density of PUUV carrying bank voles is highest in the late autumn of the increase and peak years, thus clearly explaining the multiannual and seasonal patterns in humans. However, PUUV seroprevalence in bank voles in the boreal zone is not density-dependent; it is primarily seasonal [47]. PUUV seroprevalence (maternal antibodies excluded) starts to increase in late summer–autumn in young animals. With the aging of voles and more time, prevalence increases in overwintering voles until spring–early summer and can eventually approach 80%–90% in old, overwintered animals before they disappear. This seasonal pattern occurs both in low and high densities. Of course, the absolute density of infectious voles is highest in late autumn of a peak population, but the prevalence is seasonal. Even if the prevalence increases until next summer, the actual density of the vole population declines from autumn onwards.

#### 2.1.3. Boreal vs. Temperate Patterns

The boreal pattern clearly differs from the temperate one. In the north, vole population fluctuations are to a great extent driven by small specialist predators [44,45,48], while bank vole outbreaks in the temperate zone are due to masting (seed crops of deciduous trees) [49,50,51]. In the north, it is top-down regulation; in the temperate, bottom-up system. The fluctuations may superficially look similar, but the underlying causes are different. In the temperate, the highest transmission seems to be in breeding voles in spring–summer, while in the boreal zone, the highest transmission and peak density of infected voles is among nonbreeding subadults in late autumn–early winter. We have coined the expression biome-specific epidemiology to emphasize that the epidemiological patterns vary geographically [52]. Consequently, there are clear differences in human NE epidemiology. For instance, the seasonality of incidence is very different: in the boreal zone, late autumn–early winter is the epidemic season [5,42], with the exception of people spending summer holidays in their cottages in July, resulting in a peak for city people in August, while in the temperate zone NE peaks in late spring–summer [53]. In Finland, the time lag from vole peak density in autumn to high human incidence is 2–3 months [46], and that includes the long incubation time in humans, 2–6 weeks.

#### 2.1.4. Geographic Synchrony: Local and National Statistics

PUUV and NE are very common almost everywhere in Finland within the distribution range of the bank vole, which covers the whole country except for the very northernmost parts. Unlike the temperate zone, where climate-induced masting events occur synchronously over large areas [50], bank vole cycles in Finland are not synchronous over the whole country [54]. There can be several nonsynchronous cyclic regions. In addition, the configuration of the synchronous regions changes with time, and that affects the annually reported incidence patterns, i.e., the annual number of cases that come from a restricted area. Thus, the national incidence curve can look quite different from that of local patterns. In other words, pooling the data for the whole country can hide the local cyclic patterns.

#### 2.1.5. Landscape Structure

The commonness of bank vole and PUUV all over Finland is, at least partly, due to the large homogenous taiga forests where the dispersal of the bank voles and PUUV is easy. This landscape structure is quite different from many areas in temperate Europe, where bank vole habitats are much more fragmented, and the dispersal of voles and viruses is less efficient. In a highly endemic area in Central Finland, we found that within 25 km^2^, both the bank voles and PUUV in them were genetically quite homogeneous [38,55], indicating strong dispersal and spread at this scale. Furthermore, in the increase phase of the density cycle, PUUV spreads with bank voles from practically zero occurrences in the deep crash to almost the whole landscape within a year.

#### 2.1.6. Impact of Forest Management

Forest management is very efficient in Finland, and the proportions of various successional stages (clear-cuts and young plantations, seedling stages, young forests, and old forests) are about the same. From the perspective of the bank vole, all of this, however, is “forest”, and PUUV is found in all successional stages, not only in mature forests [56]. This also contributes to the commonness of PUUV and NE in Finland.

Bank voles encounter other vole species such as field voles *Microtus agrestis* and *Sorex* shrew species in the same habitats. Dilution impact has often been invoked in these multispecies small mammal communities. Could the other species affect the contact rates in bank voles (when density was controlled for) that could reduce the prevalence of PUUV (direct dilution), or could other interspecific interactions affect the PUUV transmission in bank voles (apparent dilution)? Voutilainen et al. [56] found that in spring, when all animals were breeding and more or less territorial, real dilution occurred, while in autumn, when most animals were nonbreeding, nonterritorial juveniles and subadults, no dilution was observed. Seasonality and population structure and demographic features have to be considered in dilution analyses.

#### 2.1.7. Indirect Transmission: Virus Survival in Rodent Excreta

Hantaviruses do not have vectors, and therefore hantaviruses during their evolution have adapted to the prevailing indirect transmission and to the long bottle-neck phases in density during cyclic deep crash phases in the north. As told above, most of the transmission takes place in late autumn–winter when the vole populations consist of subadults, nonbreeding animals without real territorial behavior and no fighting. When Kallio et al. [57] experimentally studied the survival of infectiousness of PUUV in the excreta outside the host, the results surprised us: PUUV remains infectious for 2 weeks at room temperature. Further experiments in winter under the snow preliminarily suggest that survival in winter can be several weeks (Sironen et al. unpublished). Because hantavirus infection in humans is mostly through inhalation via aerosols, the long survival of PUUV in the excreta is an important factor in risk assessment, rodent management, and diagnosis. Even if rodents are temporally controlled, the infectious virus can survive in excreta dust for weeks in cottages, storage buildings, and woodsheds. The northern cold winter conditions further improve the survival of PUUV.

#### 2.1.8. Viral Shedding by Bank Voles

One of the central questions in risk management of hantavirus epidemiology is the duration of virus shedding by the rodent host. Hardestam et al. [58] found in their laboratory experiments that shedding lasted for about 2 months. However, it could be speculated that in suitable laboratory conditions with plentiful food voles could allocate more resources to immune defense than in nature. Voutilainen et al. [47] followed individually marked wild bank voles in the field with a 4-day live-trapping period per month for seven years. During the last two years, blood, urine, feces, and saliva from voles were collected monthly [59]. The infection time could be determined on the basis of seroconversion. In the field, wild bank voles did shed the virus for the rest of their lives once infected. Some individuals could be followed by shedding for 8 months. We hypothesize that limited resources of wild voles in nature limit the allocation of resources into immune defense, thus allowing shedding to continue. The long survival of PUUV outside the voles and life-long shedding probably contribute to the maintenance of PUUV in strongly cyclic bank vole populations in spite of the regular density bottlenecks.

#### 2.1.9. The Importance of Maternal Antibodies

All mammals deliver maternal antibodies to their young. In the bank vole, maternal PUUV antibodies can be seen in the young for 60–80 days (maternal IgG in young bank voles) [60], and, in fact, the protection could last even longer. Kallio et al. [61] suggested that the high prevalence of PUUV in bank voles in the spring of the peak year results in a high proportion of young individuals with maternal antibodies. This could delay the infection process in the summer of the peak year. Voutilainen et al. [47] found that in the summer of the peak year, at least 30% of young bank voles had maternal antibodies. Further, Rejniers et al. [62] modeled the role of PUUV maternal antibodies, spacing behavior, and territorial/home range dynamics of bank voles and tried to distinguish between these various components in determining the PUUV spread in a bank vole population. The modeling was based on the intensive 7-year monthly data [47]. The difference in PUUV dynamics found between summers of increase and peak years was best explained by the impact of maternal antibodies. This explains a “mystery” found in human epidemiology in Finland. Sometimes there are more human cases in the autumn of the increase year, sometimes in the peak year. This is in spite of the fact that the vole density is higher in the peak year. The explanation probably is that if most of the young, borne by old overwintered PUUV positive females in the spring of the peak year, have maternal antibodies, that could delay the transmission in young voles by 2–3 months, which could be reflected in lower human incidence in the autumn.

#### 2.1.10. Impact of PUUV on the Bank Voles

Hantaviruses have coevolved with their hosts and are generally thought to have little or no impact on host survival or reproduction. Still, the common question is: Do hantaviruses have an impact on the host? Kallio et al. [63] examined the effect of PUUV infection on the winter survival of bank voles on the 55 island experiments in the large Lake Konnevesi in Central Finland. They found that voles that were PUUV seropositive in the autumn had a lower overwinter survival rate than antibody-negative ones. Our unpublished work (Trebaticka et al.) indicates that PUUV affects the energy metabolism of bank voles in cold temperatures and high activity.

Does PUUV impact the fitness of female bank voles: i.e., the probability of breeding, reproductive effort, and mother and offspring condition? Kallio et al. [64] found that infected young females had a significantly higher, and old individuals lower, likelihood of reproducing than uninfected animals during the middle of the breeding season. PUUV infection was related to the mother’s body condition. Infected mothers were in poorer condition than uninfected mothers in the early breeding season, but they were in better condition than uninfected mothers during the middle of the breeding season. Offspring body condition was positively associated with the mother’s body condition, which, in turn, was related to the PUUV infection status of the mother. The infection may have complex effects that are dependent on the age of the individual and the time of the breeding season.

#### 2.1.11. Other Hantaviruses in Finland

Finland has the highest documented incidence of diagnosed hantavirus disease globally: in a population of 5.5 million, 1000–3000 cases of PUUV-HFRS occur annually [3,4]. For this reason, PUUV and NE have dominated the Finnish hantavirus research.

Notably, the Saaremaa virus carried by striped field mouse (*Apodemus agrarius*) and Seoul virus carried by rats [4] have not been carefully studied for infections in rodents or humans in Finland to provide conclusive evidence. The only observation of Dobrava virus strain Kurkino (originally described as Saaremaa virus) was based on serology and could not be confirmed with PCR.

Recently, however, new research on hantaviruses in *Sorex* shrews has been performed [65,66]. Genetic analyses revealed that four soricomorph-borne hantaviruses circulate in Finland, including Boginia virus (BOGV) in water shrew (*Neomys fodiens*) and Asikkala virus (ASIV) in pygmy shrew (*Sorex minutus*). Interestingly, on two study sites, common shrews (*Sorex araneus*) harbored strains of two different hantaviruses: Seewis (SWSV) virus and a new distinct, genetically distant virus (Altai-like virus), which cluster together with viruses in the basal phylogroup I of hantaviruses. This is the first evidence of the coexistence of two clearly distinct hantavirus species circulating simultaneously in one local host species population.

The results suggest that Finnish SWSV recolonized Finland from the east after the last ice age 12,000–8000 years ago and then subsequently spread along emerging land bridges toward the west or north with the migration and population expansion of its host. The difference between the L- and S-segment phylogenies implied that reassortment events play a role in the evolution of SWSV in Finland.

## 3. Clinical Features of NE

### 3.1. Renal Disease, A Dominant Feature in NE

The first scientific report from Finland about NE was published in 1964, successive renal biopsy findings of one patient were described [67]. In the 1970s, a Finnish group described renal biopsy findings in a series of 80 NE patients. The main findings have been summarized in the review [68]. Light microscopy showed tubular and interstitial alterations; medullary hemorrhages were found in 60% of the samples. By immunofluorescence study, focal and segmental glomerular immune deposits were found. Electron microscopy disclosed the fusion of glomerular podocyte foot processes [68]. This ultrastructural change decreases the barrier functions of the kidney and is one cause of proteinuria in NE [25].

Acute tubulointerstitial nephritis in NE was also documented in a later study, in which clinicopathologic correlations were found to be quite weak. It was concluded that in clinical work, renal biopsy is not indicated for the determination of the severity of AKI in NE [69]. Interstitial infiltration of several cell types, as well as the expression of cytokines and adhesion molecules in the peritubular area, were documented in another study [70].

Smoking is a known risk factor for PUUV infection [3,7,25]. In a large series of NE patients, we have shown that smoking is common in hospital-treated patients and that current smokers suffer from more severe AKI and inflammation than ex-smokers or never-smokers [71]. The mechanisms need further studies.

The content and dynamics of proteinuria, as well as the presence and associations of hematuria, have been reported in several studies [72]. One interesting finding was that urinary excretion of interleukin-6 (IL-6) correlates with the amount of proteinuria in NE [73]. We concluded that urinary IL-6 levels might reflect local production of the proinflammatory cytokine in the kidneys during acute disease. Recently we also reported that glucosuria on admission to hospital is relatively rare, but when present, it is a suitable marker for the severity of AKI and the overall course of the PUUV infection [74].

### 3.2. Gastrointestinal Bleedings

Many NE patients have abdominal pain, nausea, vomiting, and diarrhea [1,25]. Hematemesis and melena may sometimes also occur. In a Finnish study, a gastroscopy was performed in 10 consecutive patients, and in every case, a hemorrhagic gastropathy was observed [75]. The lesions were most prominent in the proximal part of the stomach, and in 7/10 patients, they were also found in the duodenum. A colonoscopy that was performed on one patient showed spotty hemorrhages. The entire gastrointestinal tract may thus be affected in NE. Histologically the lesions were associated with edema in the lamina propria but without inflammatory changes [75].

### 3.3. Cardiologic Findings

Acute myopericarditis was clinically diagnosed in 3/126 NE patients [76]. However, more than half of the 70 consecutive, hospital-treated NE patients had transient abnormal cardiac findings in a study in which the patients were prospectively examined using electrocardiograms (ECG) and echocardiography (ECHO) [77].

### 3.4. Hematologic Findings

Thrombocytopenia and mild bleeding tendency in NE are considered signs of disseminated intravascular coagulopathy. Elevations of prothrombin fragments and especially D-dimer indicate increased thrombin formation and marked fibrinolytic activity during the acute phase of the disease. Enhanced fibrinolysis could compensate for coagulation activity and contribute to clinical recovery [78]. A review of hematological findings is included in this Special Issue [Koskela et al. Coagulopathy in Puumala hantavirus infection. Viruses 2021, submitted].

### 3.5. Endocrinologic Findings

Many hormonal alterations are common during acute NE, and the hormone deficits of central origin correlate with the clinical severity of the infection [79]. In this study, many patients developed chronic hormonal deficiencies during a follow-up of several years [79]. In another Finnish study, however, no late-onset cases of hypopituitarism were observed [80]. A review of the central nervous system and ocular manifestations is included in this Special Issue [81].

### 3.6. Long-Term Consequences

Contrary to the worldwide well-accepted KDIGO criteria, even severe AKI associated with PUUV infection has a favorable outcome [82]. However, three to seven years after the acute NE, patients had slightly more proteinuria, higher glomerular filtration rate (GFR), and higher systolic blood pressure compared with the healthy controls [83]. A further follow-up of the patients showed that after 10 years from the infection, glomerular hyperfiltration and previously detected elevated proteinuria had disappeared [84]. It was concluded that, still, the possibility exists that NE may predispose some patients to the development of hypertension. The severity of acute PUUV infection does not predict the long-term outcome of the patients [85].

Chronic glomerulonephritis (GN) is a rare consequence of PUUV infection. We have described 12 patients in whom the nephrotic syndrome with peripheral swellings developed during the convalescent phase of NE [72]. The most common renal biopsy finding was mesangiocapillary (synonymous with membranoproliferative) GN. In all but one of the patients, the prognosis of GN was favorable. Many other infections, including bacterial or viral (hepatitis B and C) infections, are known to associate with the development of mesangiocapillary GN.

### 3.7. NE in Children

The clinical course of NE seems to be less severe in children than in adults [86]. The same conclusion was made in a systematic literature review that included 53 published studies. Children with PUUV infection rarely, if ever, need any invasive therapy [87]. No significant differences in the clinical severity of NE were observed between HLA B8-DRB1*03 positives and negatives [88]. An explanation could be the mild course of the disease in children.

## 4. Radiologic Findings

Lung and renal radiological findings and their clinical correlations have been studied in Finland, and the main findings have been summarized in a review article [88]. In about one-third of the patients, pulmonary findings occur, with pleural effusion, atelectasis, and interstitial infiltrates being the most common abnormalities. Severe findings, including alveolar edema and intense infiltrates, were found in 2%–4% of patients. The occurrence and severity of chest radiograph findings are associated significantly with the degree of AKI, leukocytosis, and thrombocytopenia [89]. Interestingly, pulmonary high-resolution computed tomography (HRCT) showed lung parenchymal abnormalities in almost all studied NE patients [90]. Pleural effusion showed the strongest association with the HLA-B*08 and DRB1*0301 haplotype [91].

Renal ultrasound has been undertaken in NE patients during the acute phase of the disease, and a control study was performed about five months later. During acute disease, the resistive index was abnormal, and fluid collections (perirenal, pleural, pericardial, ascites) were found in half of the patients [89]. Kidneys are swollen at the acute phase, as renal length decreased in every patient from the first to the second study. Abnormal ultrasound findings were associated with fluid volume overload and the degree of AKI. Using magnetic resonance imaging (MRI), it was shown that spleen enlargement is common during acute NE, and it might reflect the pooling of leukocytes in the spleen [92].

## 5. Severity Biomarkers

A number of biomarkers in NE have been studied in Finland [72]. High plasma interleukin-6 (IL-6) associates with clinically severe disease and can be used as a marker of the severity [93]. High C-reactive protein (CRP) as such does not indicate a severe form of NE, and, interestingly, high CRP may even be a protective factor for severe AKI in NE. Interestingly, CRP injection has been found protective in experimental immune-complex-mediated -nephritis [94]. A review of the biomarkers in PUUV infection is included in this Special Issue [Outinen et al. Severity biomarkers in Puumala hantavirus infection. Viruses 2021, in preparation].

## 6. Treatment

Vascular dysfunction and increased capillary leakage, sometimes associated with hypotension and even shock, are typical for hantavirus infections [3,25,95]. We have described the successful use of icatibant, a drug licensed for the treatment of acute episodes of hereditary angioedema (HAE), in two very severe cases of NE [18,19,20]. Icatibant is a synthetic polypeptide that acts as a selective antagonist for the bradykinin (BK) type 2 receptor, and it reduces increased vascular permeability and inhibits vasodilatation. Interestingly, increased vascular permeability during experimental hantavirus infection can be prevented with drugs that block BK binding [96]. Clearly, more extensive studies on icatibant are needed.

## 7. Concluding Remarks

Bank vole PUUV is an interesting and widely studied model of host-pathogen interactions from many different angles, from biomic impacts on host and pathogen dynamics and human epidemiology, viral ecology, and viral processes in hosts. All of this emphasizes the necessity to perform comparative studies in varying environmental conditions.

The pathogenesis of NE and other hantavirus diseases is complicated in many respects. The incubation time is long, and when clinical symptoms appear, the virus has already established a general infection in multiple tissues [3,25]. This complicates the application of antiviral treatment. However, measures to counteract key events in pathobiology offer a promising strategy, as shown by the use of icatibant to counteract vascular leakage.

Notably, however, reinfections do not exist. Thus, hantavirus vaccines are a potential possibility. However, vaccines acceptable by “western” standards are yet to come.

## Data Availability

Not applicable.
